# RESPONDING TO THE HEALTH CARE ENVIRONMENT: DEVELOPMENT OF CBO NETWORKS TO ADDRESS SOCIAL DETERMINANTS OF HEALTH

**DOI:** 10.1093/geroni/igad104.0841

**Published:** 2023-12-21

**Authors:** Beth Blair, Suzanne Kunkel, Abbe Lackmeyer, Robert Graham, Marisa Scala-Foley

**Affiliations:** USAging, Washington, District of Columbia, United States; Miami University, Oxford, Ohio, United States; Miami University, Oxford, Ohio, United States; Miami University, Oxford, Ohio, United States; USAging, Washington, District of Columbia, United States

## Abstract

AAAs, with their wide array of community partnerships and ability to respond to local needs and context, are an important connection between social and health care. Recent research describes the impact of AAA services and partnerships on population health, and AAAs are increasingly contracting with health care systems and payers to provide a variety of supportive services that enable older adults to live at home and in the community. To better meet the needs of health care partners, and to maximize their service reach and impact, AAAs and other community-based organizations (CBOs) are increasingly forming networks to contract with health care entities. Data from the 2021 CBO-Health Care Contracting Survey shows that 44 percent of AAAs are part of a network that provides contracted services to health care entities, but little is known about how these networks are formed and sustained. Findings from a new qualitative study on CBO networks, which consisted of 23 semi-structured interviews with network leads (also known as community hubs) and member organizations, describe how AAAs and other CBOs are developing networks and contracting with health care entities. This session will share findings from the qualitative study to describe the opportunities and ongoing challenges of network participation. Networks allow CBOs to serve more people and contract with health care partners to provide integrated care, but ongoing challenges include lack of data infrastructure and financial viability for members that may have fewer clients to serve. This session will also discuss the policy implications of these findings.

